# Differentiation of Retinal Ganglion Cells and Photoreceptor Precursors from Mouse Induced Pluripotent Stem Cells Carrying an Atoh7/Math5 Lineage Reporter

**DOI:** 10.1371/journal.pone.0112175

**Published:** 2014-11-17

**Authors:** Bin-Bin Xie, Xiang-Mei Zhang, Takao Hashimoto, Amy H. Tien, Andrew Chen, Jian Ge, Xian-Jie Yang

**Affiliations:** 1 Zhong-Shan Ophthalmic Center, Sun Yat-Sen University, Guangzhuo, China; 2 Jules Stein Eye Institute, University of California Los Angeles, California, United States of America; 3 Molecular Biology Institute, University of California Los Angeles, California, United States of America; NIH/NEI, United States of America

## Abstract

The neural retina is a critical component of the visual system, which provides the majority of sensory input in humans. Various retinal degenerative diseases can result in the permanent loss of retinal neurons, especially the light-sensing photoreceptors and the centrally projecting retinal ganglion cells (RGCs). The replenishment of lost RGCs and the repair of optic nerve damage are particularly challenging, as both RGC specification and their subsequent axonal growth and projection involve complex and precise regulation. To explore the developmental potential of pluripotent stem cell-derived neural progenitors, we have established mouse iPS cells that allow cell lineage tracing of progenitors that have expressed Atoh7/Math5, a bHLH transcription factor required for RGC production. These Atoh7 lineage reporter iPS cells encode Cre to replace one copy of the endogenous Atoh7 gene and a Cre-dependent YFP reporter in the ROSA locus. In addition, they express pluripotent markers and are capable of generating teratomas in vivo. Under anterior neural induction and neurogenic conditions in vitro, the Atoh7-Cre/ROSA-YFP iPS cells differentiate into neurons that co-express various RGC markers and YFP, indicating that these neurons are derived from Atoh7-expressing progenitors. Consistent with previous in vivo cell lineage studies, the Atoh7-Cre/ROSA-YFP iPS cells also give rise to a subset of Crx-positive photoreceptor precursors. Furthermore, inhibition of Notch signaling in the iPSC cultures results in a significant increase of YFP-positive RGCs and photoreceptor precursors. Together, these results show that Atoh7-Cre/ROSA-YFP iPS cells can be used to monitor the development and survival of RGCs and photoreceptors from pluripotent stem cells.

## Introduction

The neural retina is a component of the central nervous system and plays an essential role in the acquisition and processing of visual information. The mature retina consists of distinct neuronal cell types derived from a common pool of neural progenitor cells during development [1]–[3]. Many blinding diseases, including the prevalent age-related macular degeneration (AMD) and glaucoma, involve the permanent loss of retinal cells, especially the light-sensing photoreceptors or the centrally projecting retinal ganglion cells (RGCs). Despite the evolutionarily conserved neurogenic process and anatomic structure of vertebrate retinas, the spontaneous repair and regenerative capacity of the mammalian retina appears limited compared to teleosts and amphibians [4]–[7]. Therefore, de novo production of distinct retinal neurons, especially photoreceptor cells and RGCs, for the purposes of repairing damaged retinas as well as enabling disease mechanism studies remains a high priority.

RGCs are the first neuronal cell type to emerge in the developing vertebrate retina, and remain a minor cell population [8], [9]. The production of RGCs from the retinal primordium is stringently controlled by cell-intrinsic transcription factors and influenced by cell-extrinsic signals. The basic-helix-loop-helix (bHLH) transcription factor Atoh7/Math5 plays a critical role in RGC fate specification. In the absence of Atoh7, the majority of RGCs fail to develop in the mouse retina [10], [11]. The subsequent differentiation of postmitotic RGCs requires the high-mobility-group (HMG) domain transcription factors Sox4 and Sox11 [12], and the POU-domain transcription factor Pou4f/Brn3 [13]–[15]. In the early neurogenic retina, Atoh7 mRNA is expressed by a subset of progenitors [16]. The homeobox gene Pax6, which participates in eye primordium determination and controls the pluripotency of retinal progenitor cells, positively regulates Atoh7 transcription [17]–[19]. In addition, Atoh7 expression and its activity are influenced by the bHLH factors Neurog2 and Hes1 [20], [21]. Cell lineage tracing studies have shown that the progeny of Atoh7-expressing progenitors also give rise to a subset of early born cone photoreceptor cells [22], [23]. In the vertebrate retina, RGC production is negatively regulated by Notch signaling and a number of RGC-derived secreted factors, including GDF11, Sonic Hedgehog (Shh), and VEGF [24]–[30]. Consistently, genetic disruption of Notch1, GDF11, or Shh signaling increases Atoh7 expression and enhances RGC and cone photoreceptor genesis [26], [28], [29], [31]–[33].

In recent years, several groups have established protocols that permit the development of retinal neurons from embryonic stem (ES) cell or induced pluripotent stem (iPS) cell cultures [34]–[37]. As a strategy to track stem cell differentiation, fluorescent reporters have been used to monitor ocular tissue and retinal neuron development from the pluripotent cell state. Mouse ES cells with a GFP reporter replacing the retinal specific homeobox gene Rax/Rx have allowed the enrichment of retinal progenitor cells [36]–[38], and have been used successfully to demonstrate the self-organizing property of the developing optic cup and the differentiation potential of the neural retina [39]. In addition, a virally encoded fluorescent reporter driven by a photoreceptor-specific promoter has been used to enrich photoreceptors derived from human iPS cells [35]. Here, we report the establishment of mouse iPS cells that encode Cre in place of one copy of the Atoh7 gene and a Cre-dependent fluorescent reporter inserted into the ROSA locus. We show that these iPS cells express the fluorescent reporter under conditions promoting ocular tissue induction and neural differentiation, and develop into RGCs and photoreceptor precursors in vitro. These Atoh7 reporter iPS cells can thus be used to monitor a subset of retinal neuronal lineages derived from pluripotent stem cells, and to study the development and survival of RGCs and photoreceptors.

## Materials and Methods

### Animals

The Atoh7/Math5-Cre knock-in mouse was described previously [22] and the ROSA-YFP Cre reporter mouse [40] was obtained from the Jackson Laboratory (stock number 003310). We crossed these two mouse lines to generate double heterozygous offspring with the *Atoh7^CreKI/+^; ROSA^YFP/+^* genotype. The PCR primers used for genotyping were reported previously [26] and shown in Table S1. The animal protocols used were approved by the Animal Research Committee of University of California Los Angeles.

### Establishing mouse iPS cells

Mouse embryonic fibroblasts (MEFs) were isolated from embryonic day 13.5 (E13.5) *Atoh7^CreKI/+^; Rosa^YFP/+^* embryos and expanded in DMEM with 10% fetal bovine serum (FBS). Two lentiviruses, TetO-FUW-OSKM expressing reprogramming factors and FUW-M2rtTA [41] were prepared as described previously [42]. Equal volumes of media containing TetO-FUW-OSKM and FUW-M2rtTA viruses were used to perform four consecutive infections of MEFs over a period of 48 hours in the presence of 6 µg/ml of polybrene (Sigma). The culture medium was changed 12 hours after the last infection. Five days after transduction, MEFs were dissociated using trypsin and re-plated at a density of 1×10^5^ cells per10 cm-dish on irradiated CF1 mouse feeder cells seeded on a gelatin-coated surface. After 48 hours, the culture medium was replaced by a mouse embryonic stem cell (ESC) medium that contained Glasgow Minimum Essential Medium (GMEM), 10% KnockOut Serum Replacement (KSR), 1% FBS, 0.01 mM non-essential amino acids (NEAA), 1 mM sodium pyruvate, 1× antibiotic-antimycotic (100 units/ml of penicillin, 100 µg/ml of streptomycin, and 0.25 µg/ml of Fungizone), 0.1 mM 2-Mercaptoethanol, 10^3^ units/ml leukemia inhibitory factor (LIF), supplemented with 2 µg/ml doxycycline (Sigma). Mouse induced pluripotent stem cell (iPSC) colonies were picked manually based on morphology between 4 and 8 weeks after doxycycline induction and passaged more than ten generations under mouse ES cell culture conditions. Stably passaged Atoh7-Cre/ROSA-YFP iPS cells were further characterized as described in the text and stored in liquid nitrogen.

### Differentiation of iPS cells

Atoh7-Cre/ROSA-YFP iPS cell cultures were incubated for 5 minutes with ES dissociation solution containing 0.025% trypsin, 1 mg/ml type IV collagenase, 20% KSR, 1 mM CaCl_2_ in PBS. This step was repeated 2–3 times to lift most of the iPS cells from the dish. Floating ESC clusters were collected and plated on culture dishes coated with 0.2% gelatin in ESC medium for 30 minutes at 37°C to remove residual feeder cells. After pre-plating, the floating ESC clusters were further dissociated into single cell suspension with 0.05% trypsin [39].

To differentiate mouse iPS cells, 3 ml of dissociated iPS cells at 5.6×10^4^ cells/ml were plated into 6-well cluster low attachment plates in embryoid body (EB) formation medium containing 5% KSR, 0.01 mM NEAA, 1 mM sodium pyruvate, 1× antibiotic-antimycotic, 0.1 mM 2-Mercaptoethanol, 5 µM casein kinase inhibitor-7 (CKI-7), 5 µM SB431542 in GMEM as described previously [43]. After 24 hours, the medium was changed to retinal induction medium containing DMEM:F12 at 1∶1 with 10% FBS, N2 supplement, B27 supplement, 0.01 mM NEAA, 1 mM sodium pyruvate, 1× antibiotic-antimycotic, 5 µM CKI-7, and 5 µM SB431542. After 48 hours, the EBs were transferred to Lab-Tek chamber slides coated with 1∶50 dilution of Matrigel (BD Biosciences) and incubated overnight. The next day, the medium was changed to retinal differentiation medium, which was similar to retinal induction medium but without FBS. The medium was replaced every 2–3 days for the remainder of the culture period. DAPT, N-[N-(3, 5- Difluorophenacetyl)-L-alanyl]-S-phenylglycine t-butyl ester (Sigma), was added to cultures on day 7 to the final concentration of 10 µM when indicated.

### Alkaline phosphatase histochemistry

Cells were fixed in 4% paraformaldehyde/PBS for 2 minutes at room temperature followed by washes with PBS. The iPS cells were then incubated with alkaline phosphatase detection buffer containing 0.1 mg/ml 5-bromo-4-chloro-3-indolyl phosphate (X-Phos) and 0.25 mg/ml nitroblue tetrazolium (NBT) in the dark at room temperature as previously described [44].

### Immunohistochemistry

Immunolabeling was performed as previously described [26]. Cells were fixed in 4% paraformaldehyde/PBS for 10 minutes at room temperature followed by washes with PBS. After incubating in blocking solution containing 10% FBS, 2% goat or donkey serum, 0.1% Triton X-100, 0.02% sodium azide, fixed cells were incubated overnight at 4°C with the following primary antibodies: rabbit anti-GFP (1∶200, Invitrogen, used to detect YFP-expressing cells throughout the study), rabbit anti-Pax6 (1∶200, Millipore), rabbit anti-neurofilament 145 (NF145)(1∶750, Millipore), rabbit anti-Tubb3 (1∶100, Covance), rabbit anti-Otx2 (1∶50, Abcam), goat anti-Sox2 (1∶50, Santa Cruz Biotechnology), mouse anti-Pou4f1 (1∶100, Millipore), mouse anti-Crx (1∶100, Abnova), mouse anti-NF68 (1∶400, Sigma), mouse anti-Islet1 (1∶5, Developmental Study Hybridoma Bank). After washing extensively with 0.1% Tween in PBS, the cells were incubated with secondary antibodies conjugated with Alexa 488 or Alexa 594 (1∶500, Invitrogen). Fluorescent images were captured using a Nikon E800 microscope equipped with a SPOT II camera or an Olympus FluoView 1000 confocal microscope.

### RT-PCR

Total RNA was extracted following the RNAzol B (Tel-Test, Inc) extraction procedure, and Superscript III (Invitrogen) was then used with random priming to synthesize cDNA from 1 µg of total RNA. PCR primers used are shown in Table S1, Each PCR reaction was performed for 35 cycles and the reaction products were analyzed using 2% agarose gels.

### Teratoma formation

Dissociated Atoh7-Cre/ROSA-YFP iPS cells (1×10^5^ cells) were injected subcutaneously into each of six SCID mice. Tumors ranging from 5–10 mm in diameter were harvested after 3 weeks and fixed in 4% paraformaldehyde/PBS. A standard Hematoxlin and Eosin procedure was used to stain paraffin sections of tumor tissues.

### Flow Cytometry

Flow cytometry was carried out as described [26] with the following modifications. After trypsin dissociation, single cell suspensions were incubated with 6.2 µM live cell dye (eBioscience) for 20 minute, followed by fixation with 0.25% paraformaldelhyde/PBS for 30 minutes. After washing, the cells were permeablized with 0.1% Triton X100 and incubated sequentially with primary and secondary antibodies to detect YFP and retinal cell type markers before FACS analyses using an LSRII flowcytometer (BD Biosciences). To detect YFP signals, after binding to primary rabbit anti-GFP antibodies, cells were incubated with biotinylated goat anti-rabbit antibodies (1∶200, Vector Laboratory) followed by Alexa 488-conjugated streptavidin (1∶500, Invitrogen). The cell population positively labeled for the live cell dye was analyzed using FlowJo software (Tree Star). All FACS analyses include “negative controls” in which secondary antibodies alone were used to establish the base lines of fluorescent signals. The size of the samples ranged from 50,000 to 100,000 cells each. Data from a minimum of three independent iPS cell cultures were expressed as mean ± SE. Pairwise comparisons were performed using the Student *t*-test. Values of *p*<0.05 were considered statistically significant.

## Results

### Generation of Atoh7-Cre/ROSA-YFP iPS cells

**Figure 1:**
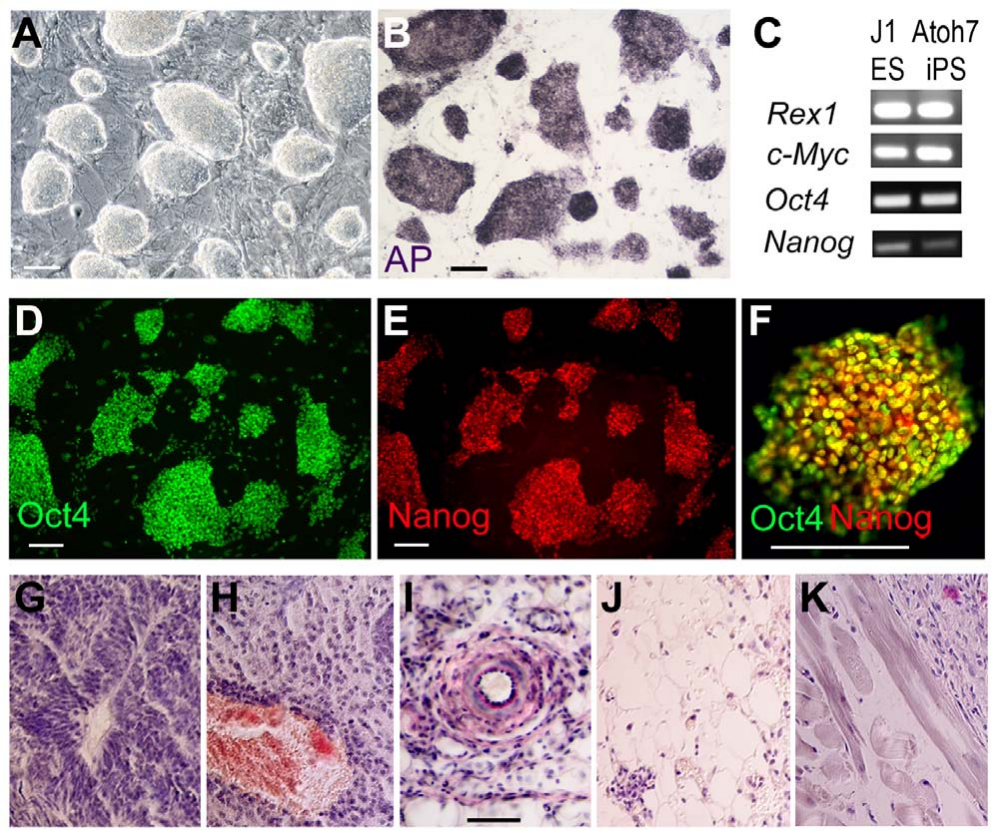
Characterization of Atoh7-Cre/ROSA-YFP iPS cells. (A) Phase contrast micrograph of Atoh7-Cre/ROSA-YFP iPS cell colonies cultured on feeder cells. (B) Alkaline phosphatase histochemical staining of Atoh7-Cre/ROSA-YFP iPS cells. (C) RT-PCR comparisons of pluripotent gene expression between mouse J1 ES cells and Atoh7-Cre/ROSA-YFP iPS cells. (D–F) Co-immunolabeling of Atoh7-Cre/ROSA-YFP iPS cells for Oct4 and Nanog proteins. A merged image of a single colony is shown in (F). (G–K) Histological staining of teratomas formed from Atoh7-Cre/ROSA-YFP iPS cells injected into SCID mice. Note the presence of multiple tissue types, including the neural epithelium (G), blood islands (H), tubular structures (I), mesenchymal tissues (J), and striated muscles (K). Scale bars, 50 µm.

Since Atoh7 is transiently expressed during retinogenesis and is no longer expressed by differentiated RGCs, we sought to establish iPS cells that will allow us to monitor Atoh7 expression as well as trace the Atoh7 lineage, including mature RGCs. We crossed heterozygous mice in which one allele of Atoh7 had been replaced by Cre (Atoh7^Cre-KI^) [22] with homozygous ROSA-YFP reporter mice [40], which encodes a Cre-dependent YFP-expression cassette, to obtain Atoh7^Cre-KI/+^; ROSA^YFP-KI/+^ double heterozygotes. As expected, at embryonic day 13.5 (E13.5) the double heterozygous retinas showed intense YFP signals in the inner retina, where postmitotic RGCs resided (Fig. S1). In addition, a subset of cells in the neuroblast layer expressed a lower level of YFP (Fig. S1). The control retinas with Atoh7^+/+^; ROSA^YFP-KI/+^ or Atoh7^Cre-KI/+^; ROSA^+/+^ genotypes did not show detectable YFP signals by immunocytochemistry (data not shown). Fibroblasts of Atoh7^Cre-KI/+^; ROSA^YFP-KI/+^ double heterozygous embryos were harvested and co-infected with a Tet-inducible lentivirus (tetO-FUW-OSKM) expressing the four Yamanaka reprogramming factors Oct4, Sox2, Klf4, and cMyc, and a lentivirus expressing rtTA [41]. After transduction with the viruses, doxycycline was added to the medium to induce the expression of the reprogramming factors, and cell colonies that acquired mouse embryonic stem (ES) cell morphology were selected and passaged under mouse ES culture conditions (Fig. 1A). These established cell lines, referred to herein as Atoh7-Cre/ROSA-YFP iPS cells, expressed a number of ES cell markers similar to those observed in the control mouse J1 ES cell line, including alkaline phosphatase, Nanog and Oct4 (Fig. 1B–F). In addition, these Atoh7-Cre/ROSA-YFP iPS cells underwent repeated freeze-and-thaw cycles after storage in liquid nitrogen, and were passaged multiple times without changing ES cell morphology and markers when cultured. To further examine the pluripotency of these MEF-derived iPS cells, we implanted the Atoh7-Cre/ROSA-YFP iPS cells into immune-deficient SCID mice. All transplantations (N = 6) into SCID mice resulted in the formation of teratomas (Fig. 1G–K), demonstrating the pluripotent nature of these iPS cells.

### Production of retinal progenitors from Atoh7-Cre/ROSA-YFP iPS cells

**Figure 2:**
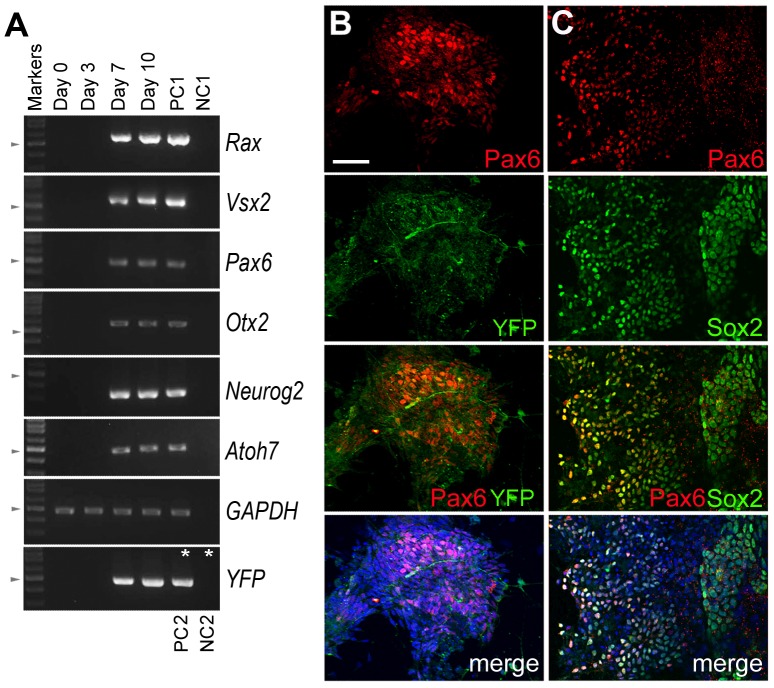
Expression of neural progenitor markers in Atoh7-Cre/ROSA-YFP iPS cultures. (A) RT-PCRs detect transcripts of genes expressed by retinal progenitor cells. Positive controls were 1 µg cDNA of P0 mouse retina as PC1, and HEK cells infected with a lentivirus expressing GFP as PC2 (*). Negative controls were total RNAs from iPS cells without reverse transcriptase as NC1, and non-infected HEK cells as NC2 (*). Arrowheads indicate the position of the 500 bp size marker. (B, C) Confocal images of immunolabeled Atoh7-Cre/ROSA-YFP iPS cell-derive EB cultures at day 7 are shown. (B) YFP-positive neurite extension, and YFP and Pax6 co-expression in a subset of cells were detected. (C) Pax6 was detected in some but not all Sox2 positive cells. The merged images also include DAPI labeling (blue) of cell nuclei. Scale bar, 50 µm for all panels.

To evaluate the developmental potential of the Atoh7-Cre/ROSA-YFP iPS cells, we used the previously described anterior neuro-induction approaches that involve manipulation of Wnt and TGFβ/BMP signals [36]. The iPS cells were allowed to spontaneously aggregate into embryoid bodies (EBs) and cultured in the presence of the TGFβ inhibitor SB431542 and the Wnt inhibitor “casein kinase inhibitor-7” (CKI-7) to promote the anterior ventral neural fate, thus enhancing formation of the eye field. The EBs were then attached to coated surfaces and cultured further in a retinal differentiation medium [43]. At day 7 from the onset of iPS cell-derived EB formation, transcripts of several homeobox genes normally expressed by retinal progenitor cells, including *Rax/Rx, Pax6, Vsx2, Otx2*, were detected (Fig. 2A). In addition, numerous Pax6-positive cells were observed in the EB cultures; however, only a subset of the Pax6-positive cells co-expressed Sox2, which is also expressed by retinal progenitor cells (Fig. 2B, C). The Atoh7-Cre/ROSA-YFP iPS cells-derived EBs also expressed the neurogenic bHLH genes Neurog2 and Atoh7 by day 7 (Fig. 2A), indicating the onset of neurogenesis at this time point in culture. Furthermore, we detected both YFP transcript and protein by day 7 (Fig. 2A, B), indicating expression of the YFP reporter under the control of Cre from the Atoh7 locus.

**Figure 3:**
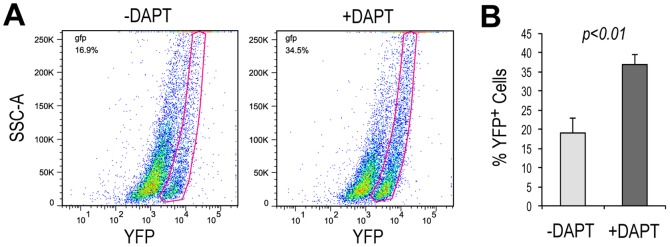
Effects of γ-secretase inhibitor DAPT on production of the Atoh7 lineage cells. (A) Representative FACS profiles for Atoh7-Cre/ROSA-YFP iPS cell cultures with or without DAPT at day 14. DAPT was added to the cultures at day 7. Red lines indicate gating for YFP-positive cells. (B) FACS quantification of YFP-positive cells in the Atoh7-Cre/ROSA-YFP iPS cell cultures at day 14. N = 4, the significant *p* value is shown.

Since Notch signaling is known to influence retinal progenitor cell proliferation and differentiation, we tested whether the γ-secretase inhibitor DAPT, which inhibits Notch signaling, affected production of YFP-positive cells in the Atoh7-Cre/ROSA-YFP iPS EB cultures. Quantitative analyses using flow cytometry showed that DAPT treatment significantly increased the percentage of YFP-positive cells from 19.0±3.8% to 36.9±2.7% by day 14 (Fig. 3), indicating that the production of cells from the Atoh7-expressing lineage was enhanced when Notch signaling was inhibited.

### Differentiation of RGCs from Atoh7-Cre/ROSA-YFP iPS cells

**Figure 4:**
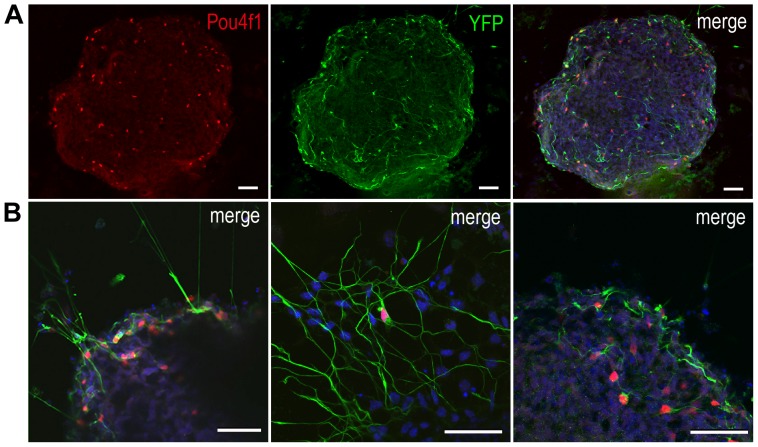
Expression of YFP and retinal ganglion cell marker Pou4f1 in Atoh7-Cre/ROSA-YFP iPS cell cultures. Confocal images show Atoh7-Cre/ROSA-YFP iPS cell cultures at day 12. (A) A representative EB co-labeled for YFP (green) and Pou4f1 (red), and also stained by DAPI (blue). (B) Expanded views showing merged images of YFP (green), Pou4f1 (red), and DAPI (blue) from different EBs at day 12. Scale bars, 50 µm.

**Figure 5:**
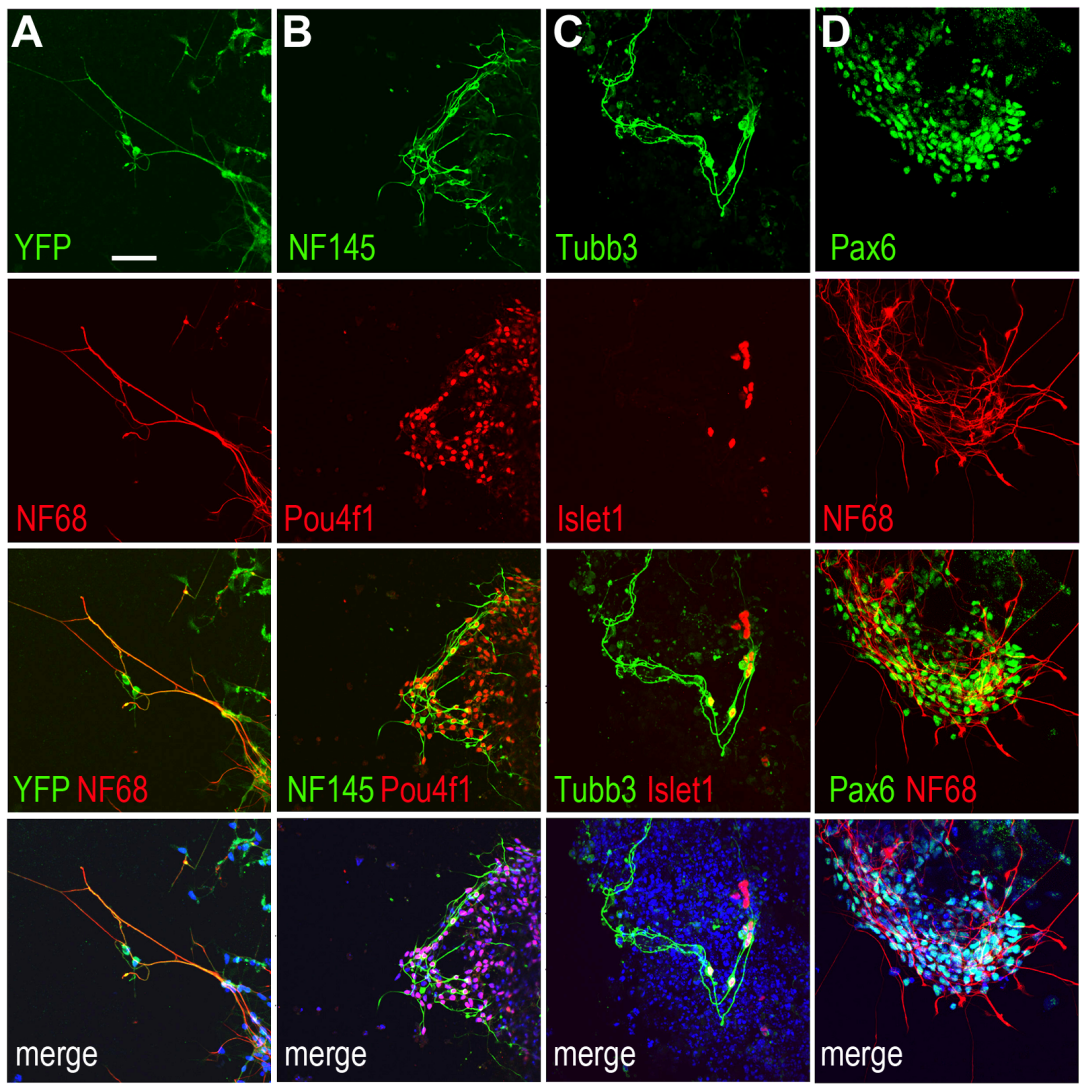
Differentiation of retinal ganglion cells from Atoh7-Cre/ROSA-YFP iPS cells. Confocal images show Atoh7-Cre/ROSA-YFP iPS cell cultures at day 12 co-immunolabeled for YFP and/or various neuronal markers, including (A) YFP and NF68; (B) Pou4f1 and NF145; (C) β-Tubulin (Tubb3) and Islet1; (D) Pax6 and NF68. The bottom panels show merged images including DAPI (blue) staining of nuclei. Scale bar, 50 µm for all.

We next examined whether Atoh7-Cre/ROSA-YFP iPS cells-derived EBs could produce RGCs. On day 12 of the EB culture, immunocytochemistry and confocal imaging revealed YFP-positive cells with extensive neurites and intense nuclear labeling for Pou4f1/Brn3a, a transcription factor involved in RGC differentiation (Fig. 4), demonstrating the production of authentic RGCs from Atoh7-expressing progenitor cells. In addition, we detected co-labeling of YFP and the low molecular weight neurofilament (NF68), another marker expressed by postmitotic RGCs (Fig. 5A). Furthermore, we observed co-expression of several other RGC markers, including Pou4f1 and the intermediate molecular weight neurofilament NF145, Tubb3/β-tubulin and Islet-1, as well as NF68 and Pax6 (Fig. 5B–D; Fig. S2). These data demonstrate that RGCs are produced in Atoh7-Cre/ROSA-YFP iPS cells-derived EBs, especially from the Atoh7-expressing lineage.

**Figure 6:**
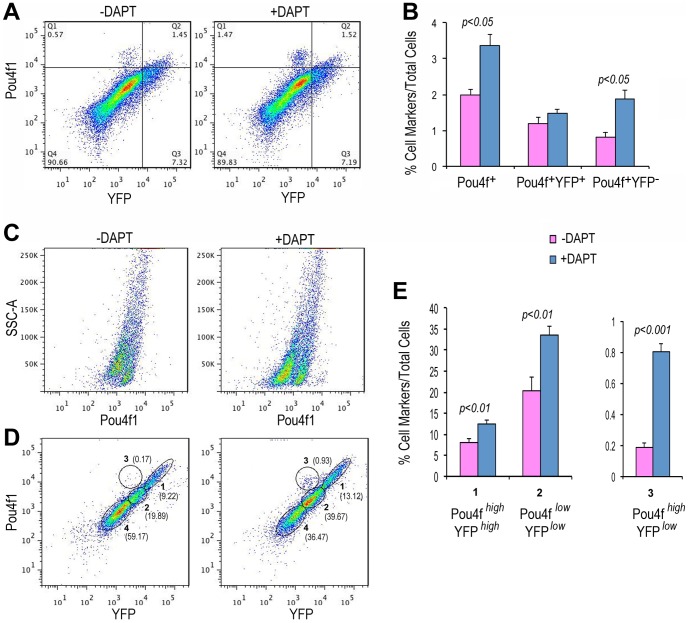
Quantitative analyses of retinal ganglion cell differentiation from Atoh7-Cre/ROSA-YFP iPS cells. (A) Typical FACS profiles show effects of DAPT on YFP and Pou4f1 markers at day 10. (B) Bar graphs show FACS quantification at day 10 of total Pou4f1+ (Q1 and Q2), Pou4f1^+^YFP^+^ double (Q2), and Pou4f1^+^ YFP^−^ (Q1) cells. N = 3, Significant *p* values are indicated. (C, D) Representative FACS profiles show effects of DAPT on Pou4f1 and YFP at day 14. Different populations of cells shown in (D) are designated as Pou4f1^high^ YFP^high^ (group 1), Pou4f1^low^ YFP^low^ (group 2), and Pou4f1^high^ YFP^low^ (group 3). (E) Bar graphs show quantification of group 1–3 cells shown in (D) under the influence of DAPI. N = 4, significant *p* values are indicated.

Previous studies have shown that inhibiting Notch signaling can enhance RGC production from early retinal progenitor cells [31]–[33]. We thus tested whether inclusion of DAPT would enhance RGC development in Atoh7-Cre/ROSA-YFP iPS cell cultures. Quantification using flow cytometry showed that the addition of DAPT at EB culture day 7 significantly increased the number of Pou4f1^+^ cells from 2.0±0.2% to 3.4±0.4% at day 10, but did not affect Pou4f1 and YFP double positive cells (Fig. 6A, B). By day 14 of the culture, flow cytometry analyses for Pou4f1 and YFP revealed four distinct cell populations, Pou4f1^high^ YFP^high^, Pou4f1^low^YFP^low^, Pou4f1^high^ YFP^low^, and Pou4f1^−^YFP^−^ (Fig. 6C, D). Among these populations, Pou4f1^high^ YFP^high^ cells likely represented authentic RGCs derived from Atoh7-expressing progenitors. DAPT treatment resulted in a statistically significant increase of Pou4f1^high^ YFP^high^ cells from 8.1±0.8% to 12.5±0.9% at day 14 (Fig. 6D, E). We also detected a significant increase in the Pou4f1^low^ YFP^low^ population, which may reflect the influence of DAPT on Atoh7-expressing progenitors during the 7-day DAPT treatment to generate other retinal cell types including early born photoreceptors (see below). Similar to our observations in the day 10 cultures (Fig. 6A, B), DAPT also significantly increased production of the minor Pou4f1^high^ YFP^low^ cell population in the day 14 cultures (Fig. 6E), which likely represented non-retinal neurons. These quantitative analyses, together with cell marker labeling and imaging, demonstrate that Atoh7-Cre/ROSA-YFP iPS cells can give rise to RGCs in vitro, and that DAPT treatment enhances RGC production in iPS cell-derived EB cultures.

### Production of photoreceptor precursors

**Figure 7:**
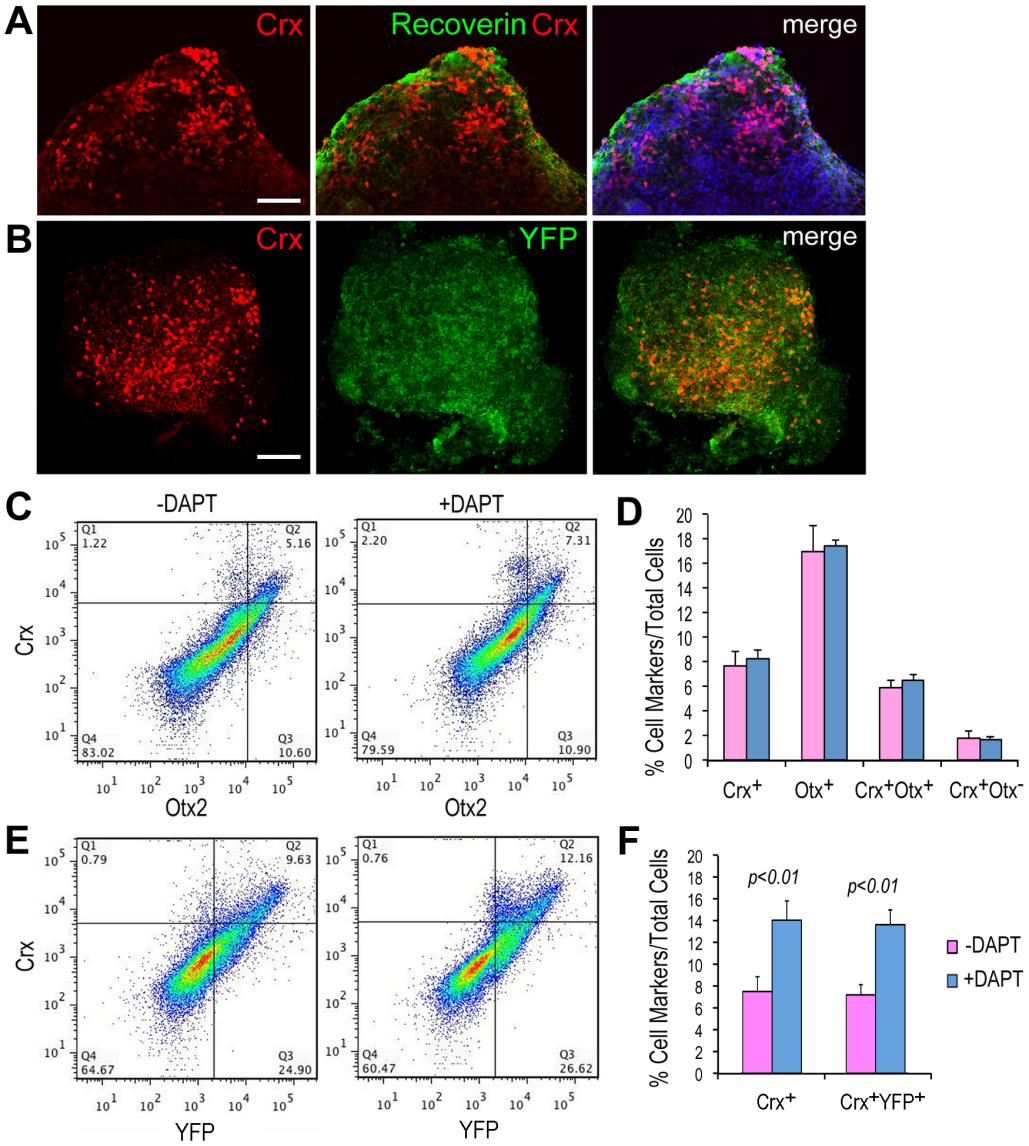
Differentiation of photoreceptor precursor cells from Atoh7-Cre/ROSA-YFP iPS cells. (A, B) Confocal images show Atoh7-Cre/ROSA-YFP iPS cell cultures at day 12 co-immunolabeled for Crx and Recoverin (A), or Crx and YFP (B). The merged image for (A) also shows DAPI labeling of cell nuclei. Scale bars, 50 µm for all. (C–F) Quantitative analyses of photoreceptor precursor differentiation in the iPS cell EB cultures. (C) Typical FACS profiles show effects of DAPT on Otx2 and Crx at day 12. (D) Bar graphs show quantification of various populations of marker positive cells with or without DAPT at day 12. (E) Typical FACS profiles show effects of DAPT on Crx and YFP at day 14. (F) Bar graphs show quantification of Crx^+^ and Crx^+^ YFP^+^ cells among total cells at day 14. N = 4, significant *p* values are indicated.

The Atoh7-expressing retinal progenitors are heavily biased toward generating RGCs as well as the early born cone photoreceptors in vivo [22], [23]. We thus examined if the established Atoh7-Cre/ROSA-YFP iPS cells could also produce photoreceptors in vitro under our culture conditions. In day 12 EB cultures, we detected strong nuclear immunofluorescent signals for the cone-rod homeobox protein Crx, which is expressed by postmitotic photoreceptor precursors (Fig. 7A, B). Confocal imaging also showed that the Crx protein expression correlated with localization of the photoreceptor protein Recoverin and cytoplasmic YFP.

To confirm that Atoh7-Cre/ROSA-YFP iPS cells can indeed give rise to Crx^+^ photoreceptor precursors, we used flow cytometry to analyze dissociated EB cells. Since the homeobox protein Otx2 promotes Crx expression [45], [46] and Notch signals suppress cone photoreceptor differentiation [31]–[33], we quantified the number of Otx2^+^ and Crx^+^ cells cultured either in the presence or absence of DAPT. We observed a minor but not statistically significant increase of Crx^+^ cells with DAPT treatment at day 12 of EB culture (Fig. 7C, D). Neither Otx2^+^ cells nor Otx2^+^Crx^+^ cells were significantly increased with DAPT treatment at day 12 (Fig. 7D). However, by day 14, DAPT treated cultures showed significantly increased Crx^+^ cells from 7.6±1.3% to 14.1±1.7%. Quantification showed that most of the Crx^+^ cells were also YFP^+^, and DAPT enhanced the Crx^+^ YFP^+^ population from 7.3±1.0 to 13.7±1.4% (Fig. 7E, F). Our results thus suggest that in these iPS EB cultures, the Atoh7-expressing lineage also generate Crx-expressing photoreceptor precursors, which can be expanded by inhibiting the Notch signal.

## Discussion

Repairing vision impairment associated with axonal damage and/or the loss of RGCs is particularly challenging, as RGCs emerge during early retinogenesis and not only connect to retinal interneurons but also extend centrally projecting axons along complex trajectory routes to establish functional visual circuitry. Moreover, the mechanisms underlying vision-impairing diseases like glaucoma and optic nerve neuropathy remain poorly understood, and have been difficult to study in vivo. It is therefore highly advantageous to establish an in vitro RGC culture system that would facilitate the discovery of disease etiologies and therapeutic treatments. In recent years, significant progress has been made in deriving retinal pigment epithelium and photoreceptors from pluripotent stem cells for therapeutic purposes [47]. However, few studies have focused on RGC differentiation [48], [49]. In this study, we have taken advantage of the available mouse genetic and cell reprogramming tools to create iPS cells that carry a molecular reporter for neurons derived from progenitors expressing Atoh7, a retinal specific gene critical for RGC development. Our results show that under favorable neural induction conditions, mouse Atoh7-Cre/ROSA-YFP iPS cells can activate the Cre reporter and recapitulate the developmental potential of the endogenous Atoh7-expressing cell lineage. These iPS cells can thus serve as a useful stem cell source for mechanistic studies of RGC and photoreceptor genesis and differentiation, as well as for studies of RGC axon trajectory, guidance, and survival.

Since we used heterozygous Atoh7^Cre-KI/+^; Rosa^YFP-KI/+^ MEFs to derive the Atoh7-Cre/ROSA-YFP iPS cells, expression of the knock-in Cre should reflect expression of the endogenous Atoh7, without affecting RGC specification. Furthermore, although Atoh7 expression in the developing mouse retina is transient, the YFP reporter expression from the ROSA locus is constitutive once the Cre-loxP recombination has occurred. Thus, the YFP reporter should mark all progeny derived from cells that have expressed Atoh7 and thus enable cell lineage tracing and monitoring. These Atoh7-Cre/ROSA-YFP iPS cells are particularly useful, as currently there are no reliable commercial anti-Atoh7/Math5 antibodies that can specifically detect Atoh7 protein in immunocytochemical labeling assays. Our characterization has shown that the YFP reporter is expressed as early as day 7 in the EB culture, correlating with the expression of a number of homeobox genes, including Rax, Pax6 and Vsx2, which are normally expressed in the eye domain and retinal primordium. In addition, the YFP reporter expression also coincides with the transcript expression of two early neurogenic bHLH factors Neurog2 and Atoh7. These temporal gene expression results are consistent with previous studies using mouse Rax^GFP-KI^ ES cells under similar culture conditions [36].

The specification of RGCs in vivo is negatively regulated by Notch signaling and a number of secreted cell extrinsic cues [24], [26], [27], [29], [30], [33]. We show that the γ-secretase inhibitor DAPT can significantly expand production of the Atoh7 lineage cells from 19% to 37% by day 14, when the majority of the YFP-labeled cells represent postmitotic neurons. This suggests that DAPT can effectively relieve the suppression of Notch and possibly other signals on RGC specification and thereby increase the number of progeny derived from Atoh7-expressing progenitors in the iPS cell EB cultures. Indeed, FACS quantification data show that DAPT treatment results in a 50% increase of the Pou4f1 and YFP double positive RGCs (from 8% to 12% by day 14). These Pou4f1 and YFP double positive cell are likely authentic RGCs as the fluorescent reporter reflects the developmental history of Atoh7 expression from its endogenous locus. Not surprisingly, DAPT treatment also enhanced a minor population (<1%) of Pou4f1-positive cells that did not express high levels of the YFP reporter. These likely represent non-retinal neurons produced in the culture, possibly cortical neurons.

Cell lineage tracing studies in vivo have shown that Atoh7-expressing progenitors also give rise to other retinal cell types, among which a large portion becomes cone photoreceptors [22], [23]. In agreement with the in vivo lineage analyses, we have also detected Crx and YFP double positive cells in the iPSC cultures, indicating that certain Atoh7 lineage-derived cells have adopted a distinct fate from RGC and become photoreceptor precursors. Interestingly, although Otx2 is an upstream positive regulator of Crx [45], [46], we did not detect any significant increase in the number of Otx2, or Otx2 and Crx double positive cells with DAPT treatment. This may reflect that Notch signaling leads to only transient enhancement of Otx2 expression in a selective population of retinal progenitors, and these cells then quickly become post-mitotic Crx-expressing photoreceptor precursors at day 12. However, DAPT treatment indeed resulted in enhanced production of Crx and YFP double positive cells (from 7% to 13% by day 14), consistent with the phenotypes of Notch1 and RBPj deletions in the retina [31]–[33]. As expected from in vivo cell lineage tracing studies [22], [23], we also observed Crx-positive photoreceptor precursors that do not express YFP, and thus were not derived from the Atoh7 lineage. Although we did not pursue the terminal differentiation of the Crx-positive photoreceptor precursors in this study, this population of Atoh7 lineage cells likely contains cone cell precursors, as their production period in vivo overlaps extensively with RGC genesis.

In summary, we have established mouse iPS cells that carry a reporter marking an important sub-neuronal cell lineage of the retina. These pluripotent Atoh7-Cre/ROSA-YFP iPS cells will be useful tools in future investigations to study ocular tissue differentiation, the onset of retinal neurogenesis, cell fate specification, as well as neuronal differentiation and survival.

## Supporting Information

Figure S1 Expression of ROSA-YFP reporter in the Atoh7-Cre knock-in retina. Fluorescent images of E13.5 eye sections from mice heterozygous for *Atoh7-Cre^KI^* and *ROSA.YFP* were immunolabeled for YFP (A) and stained with the nuclear dye DAPI (B). Scale bars in (B) represent corresponding panels in (A), 100 µm. *gcl*, ganglion cell layer; *le*, lens; *ret*, retina; *rpe*, retinal pigment epithelium; *nb*, neuroblast layer.
(TIF)

Figure S2 Expression of retinal ganglion cell markers in Atoh7-Cre/ROSA-YFP iPS cell cultures. (A, B) Confocal images show Atoh7-Cre/ROSA-YFP iPS cell derived ES cultures at day 12 co-immunolabeled for NF145 (green) and Pou4f1 (red). The merged images also show DAPI labeling of nuclei. (C) An enlarged field to show colabeling of Pou4f1 and NF145 with DAPI stained nuclei. Scale bars, 50 µm.
(TIF)

Table S1 Primers for PCR.
(DOCX)
