# Supplementary figures and images for: Differentiation of Retinal Ganglion Cells and Photoreceptor Precursors from Mouse Induced Pluripotent Stem Cells Carrying an Atoh7/Math5 Lineage Reporter
Source: PLoS One. 2014 Nov 17;9(11):e112175. doi: 10.1371/journal.pone.0112175 (PMC4234374; doi:10.1371/journal.pone.0112175)

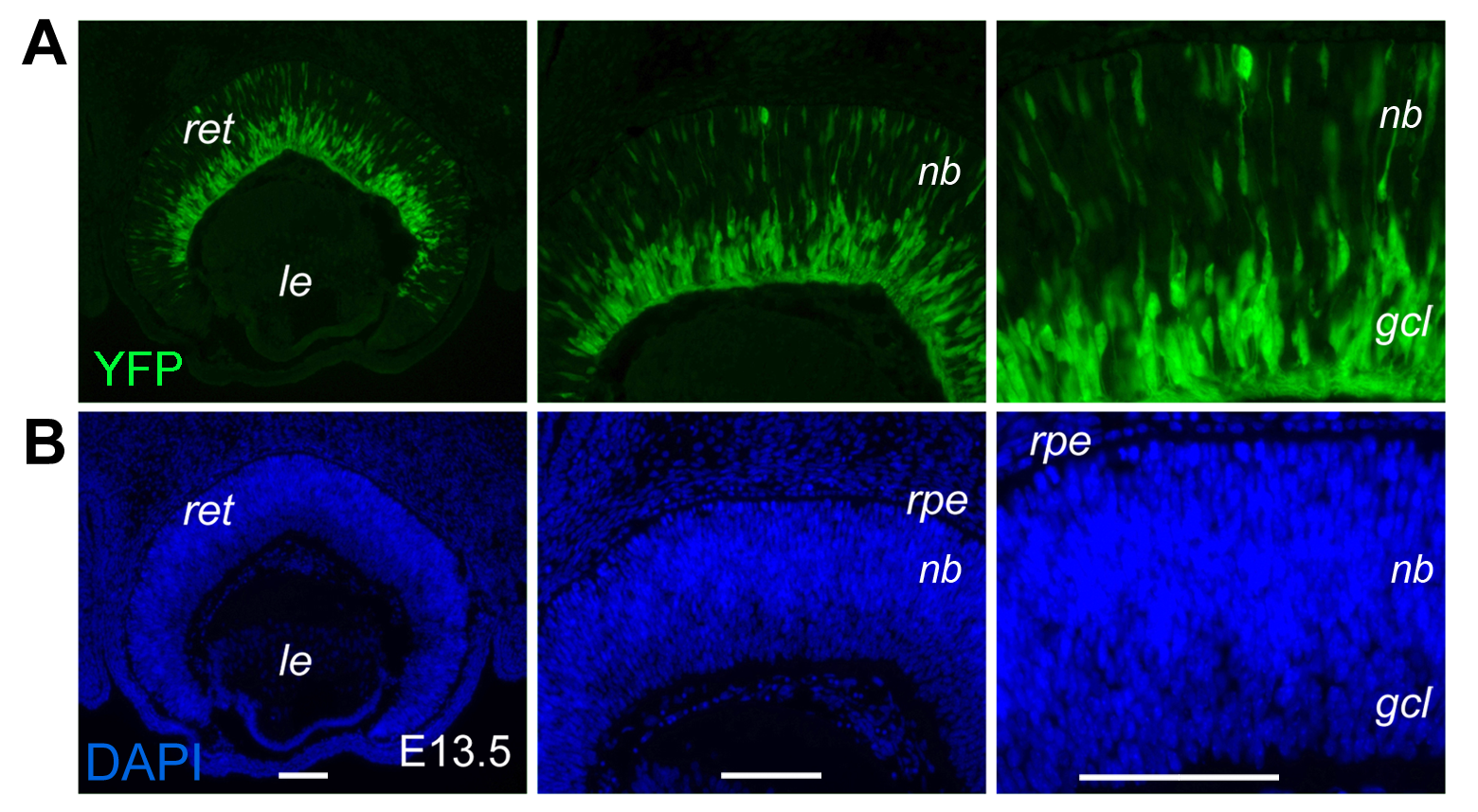

Supplement: Figure S1 — Expression of ROSA-YFP reporter in the Atoh7-Cre knock-in retina. Fluorescent images of E13.5 eye sections from mice heterozygous for Atoh7-CreKI and ROSA.YFP were immunolabeled for YFP (A) and stained with the nuclear dye DAPI (B). Scale bars in (B) represent corresponding panels in (A), 100 µm. gcl, ganglion cell layer; le, lens; ret, retina; rpe, retinal pigment epithelium; nb, neuroblast layer. (TIF) [file pone.0112175.s001.tif]

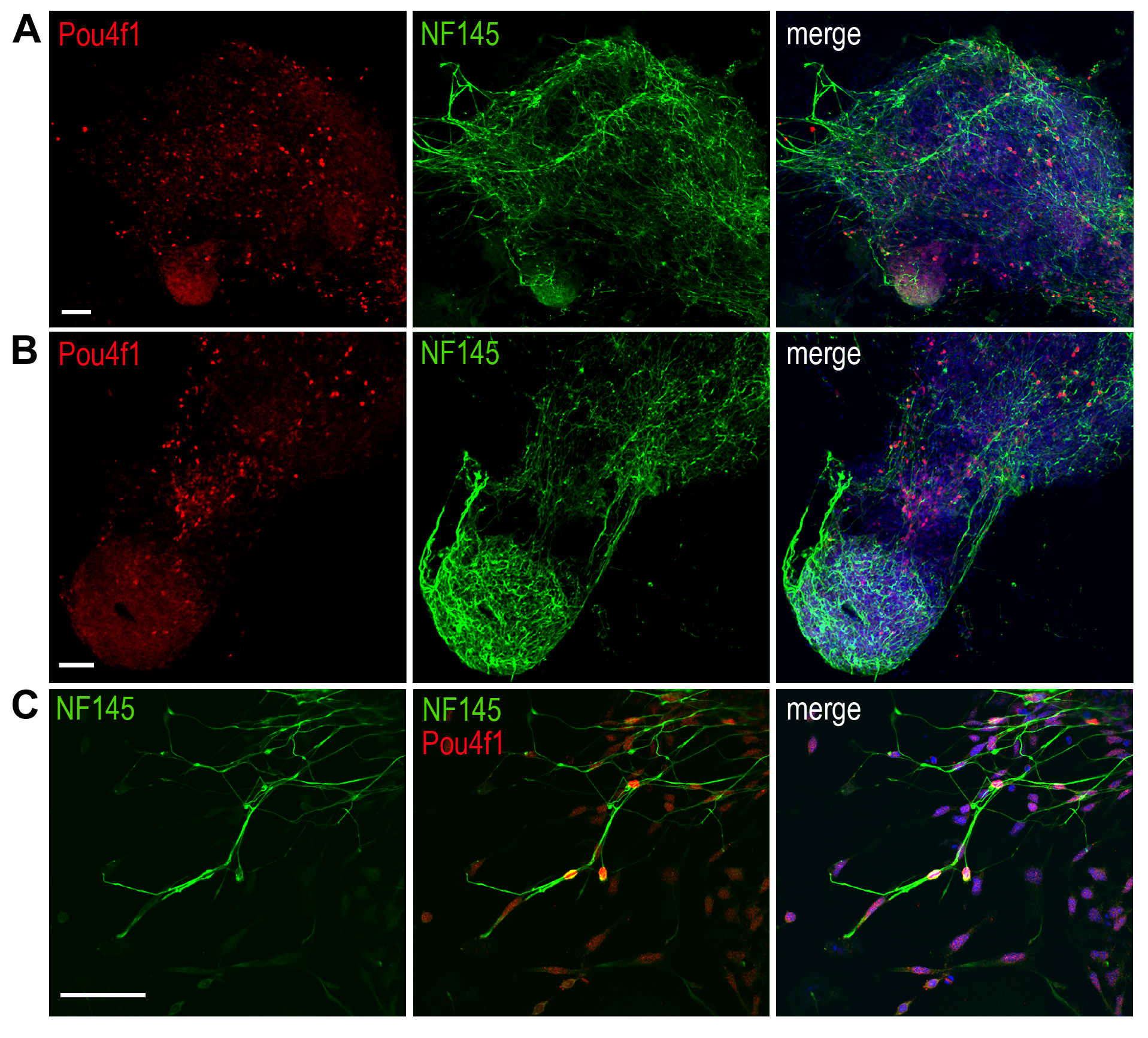

Supplement: Figure S2 — Expression of retinal ganglion cell markers in Atoh7-Cre/ROSA-YFP iPS cell cultures. (A, B) Confocal images show Atoh7-Cre/ROSA-YFP iPS cell derived ES cultures at day 12 co-immunolabeled for NF145 (green) and Pou4f1 (red). The merged images also show DAPI labeling of nuclei. (C) An enlarged field to show colabeling of Pou4f1 and NF145 with DAPI stained nuclei. Scale bars, 50 µm. (TIF) [file pone.0112175.s002.tif]
