# Supplementary material for: Differentiation of Retinal Ganglion Cells and Photoreceptor Precursors from Mouse Induced Pluripotent Stem Cells Carrying an Atoh7/Math5 Lineage Reporter
Source: PLoS One. 2014 Nov 17;9(11):e112175. doi: 10.1371/journal.pone.0112175 (PMC4234374; doi:10.1371/journal.pone.0112175)
Supplement: Table S1 — Primers for PCR. (DOCX) [file pone.0112175.s003.docx]

**Table S1. Primers for PCR**

Gene Forward Primer Reverse Primer Size (bp)

*Nanog* AGGGTCTGCTACTGAGATGCTCTG CAACCACTGGTTTTTCTGCCACCG 364

*Oct4* TCTTTCCACCAGGCCCCCGGCTC TGCGGGCGGACATGGGGAGATCC 223

*cMyc* CAGAGGAGGAACGAGCTGAAGCGC TTATGCACCAGAGTTTCGAAGCTGTTCG 228

*Rex1* ACGAGTGGCAGTTTCTTCTTGGGA TATGACTCACTTCCAGGGGGCACT 392

*Rax* GCTCTAGAAAGACGGCATCCTAGACACC CGGAATTCAGAATCCTGCAGCTTCATGG 507

*Vsx2* GCGGATCCACCAAGAAGCGTAAGAAGCG CGGAATTCAACCTTGGTGCTGTGTTCC 550

*Pax6* GCGGATCCAACAACCTGCCTATGCAACC CGGAATTCATACCGTGCCTTCTGTACGC 449

*Otx2* GCTCTAGATGCATGCAGAGGTCCTATCC CGGAATTCTCAGGTTGCTGGTTGATGG 564

*Neurog2* CGGGATCCTACAGCTGCACTTTATCGCC CGGAATTCCGCCATAGTCCTCTTTGACC 317

*Atoh7* ATCACCCCCTACCTCCCTTTC TCTCCACCTCCTGAATGACG 446

*GAPDH* CCATCAAGTCCACAACACGGTTGCTGTA GTCTTATGACCACTGTCCATGCCATCAC 468

*GFP(YFP)* AAGTTCATCTGCACCACCG TGCTCAGGTAGTGGTTGTCG 470

*Cre* GACGATGCAACGAGTGATGA AGCATTGCTGTCACTTGGTC
